# Cognitive Reserve Relates to Functional Network Efficiency in Alzheimer’s Disease

**DOI:** 10.3389/fnagi.2018.00255

**Published:** 2018-08-21

**Authors:** Marina Weiler, Raphael Fernandes Casseb, Brunno Machado de Campos, Camila Vieira de Ligo Teixeira, Ana Flávia Mac Knight Carletti-Cassani, Jéssica Elias Vicentini, Thamires Naela Cardoso Magalhães, Débora Queiroz de Almeira, Leda Leme Talib, Orestes Vicente Forlenza, Marcio Luiz Figueredo Balthazar, Gabriela Castellano

**Affiliations:** ^1^Neurophysics Group, Institute of Physics Gleb Wataghin, Cosmic Rays and Chronology Department, University of Campinas (UNICAMP), Campinas, Brazil; ^2^Neuroimaging Laboratory, School of Medical Sciences, University of Campinas (UNICAMP), Campinas, Brazil; ^3^Laboratório de Neurociências (LIM-27), Departamento e Instituto de Psiquiatria, Hospital das Clínicas da Faculdade de Medicina, Universidade de São Paulo (USP), São Paulo, Brazil; ^4^Brazilian Institute of Neuroscience and Neurotechnology (BRAINN), Campinas, Brazil

**Keywords:** fMRI, graph theory, mild cognitive impairment, neuropathology, educational measurement, network efficiency

## Abstract

Alzheimer’s disease (AD) is the most common form of dementia, with no means of cure or prevention. The presence of abnormal disease-related proteins in the population is, in turn, much more common than the incidence of dementia. In this context, the cognitive reserve (CR) hypothesis has been proposed to explain the discontinuity between pathophysiological and clinical expression of AD, suggesting that CR mitigates the effects of pathology on clinical expression and cognition. fMRI studies of the human connectome have recently reported that AD patients present diminished functional efficiency in resting-state networks, leading to a loss in information flow and cognitive processing. No study has investigated, however, whether CR modifies the effects of the pathology in functional network efficiency in AD patients. We analyzed the relationship between CR, pathophysiology and network efficiency, and whether CR modifies the relationship between them. Fourteen mild AD, 28 amnestic mild cognitive impairment (aMCI) due to AD, and 28 controls were enrolled. We used education to measure CR, cerebrospinal fluid (CSF) biomarkers to evaluate pathophysiology, and graph metrics to measure network efficiency. We found no relationship between CR and CSF biomarkers; CR was related to higher network efficiency in all groups; and abnormal levels of CSF protein biomarkers were related to more efficient networks in the AD group. Education modified the effects of tau-related pathology in the aMCI and mild AD groups. Although higher CR might not protect individuals from developing AD pathophysiology, AD patients with higher CR are better able to cope with the effects of pathology—presenting more efficient networks despite pathology burden. The present study highlights that interventions focusing on cognitive stimulation might be useful to slow age-related cognitive decline or dementia and lengthen healthy aging.

## Introduction

In 2014, the movie “Still Alice,” interpreted by the actress Julianne Moore, brought to the public the dramatic experience of someone living with Alzheimer’s disease (AD), the most common form of dementia worldwide (and yet with no means of cure or even prevention). More interesting, however, was the fact that the onset of the disease’s clinical expression was delayed by modifiable factors, such as her high intellectual level. The cognitive reserve (CR) hypothesis, in this context, has been proposed to account for the discontinuity between pathophysiological and clinical expression of AD. This hypothesis claims that individuals with higher CR can better maintain cognitive functions despite AD pathology (Stern, 2012), thus consisting in an important factor in the fight against the disease. In fact, innumerable epidemiological studies report that greater CR may postpone the clinical expression of AD (Amieva et al., 2014; Osone et al., 2015), yet accelerate the cognitive decline after its onset (Scarmeas et al., 2006; Bruandet et al., 2008).

Neuroimaging studies (Scarmeas et al., 2003; Perneczky et al., 2006; Serra et al., 2011; Liu et al., 2012; Boots et al., 2015) have consistently shown a positive relationship between brain structure/metabolism/perfusion and CR in the healthy elderly, whereas in AD patients there is an inverse relationship between them. These findings suggest that patients with higher CR are better able to cope with the brain pathology than patients with lower CR, and more pathology is needed in the former group to reach the dementia level (thus mitigating the clinical impact of the disease). Studies involving fMRI analysis (Solé-Padullés et al., 2009; Bosch et al., 2010; Arenaza-Urquijo et al., 2013a; Bozzali et al., 2015; Marques et al., 2015; Serra et al., 2017), in turn, found that healthy elders, mild cognitive impairment (MCI) and AD patients with higher CR present more activation/connectivity in brain regions than the individuals with lower CR. These interesting results have shed some light onto the issue, proposing that the healthy elderly with higher education level present increased brain efficiency, while AD patients are able to recruit compensatory mechanisms to maintain cognitive function. Because of the lack of pathophysiological data for the subjects, however, these results only support the CR hypothesis, and preclude any further assumptions on it.

The role of CR in attenuating the prejudicial effects of AD pathology in cognition has been explored in several studies involving both healthy controls (Bennett et al., 2003; Yaffe et al., 2011; Almeida et al., 2015) and patients (Roe et al., 2008; Rentz et al., 2010). Individuals with higher CR who are still cognitively normal have less chance to develop dementia, despite presenting abnormal pathological protein levels such as amyloid β (Aβ) and phospho-tau (p-tau; Soldan et al., 2013). Although the neurobiological mechanism behind this process is not known, some studies posit that a greater exposure to a cognitively stimulating environment is associated with reduced levels of the pathological proteins, protecting the individuals from developing AD-related pathology (Lazarov et al., 2005; Landau et al., 2012; Harris et al., 2015).

Among the negative impacts of the disease in a subject’s brain, is the disruption in the topological organization of the functional connectome, leading to a loss of global information integration in disease due to the randomization of the brain functional networks (Sanz-Arigita et al., 2010). Although disruption in resting-state networks has already been related to abnormal levels of cerebrospinal fluid (CSF) proteins (Aβ and 181Thr-phosphorylated tau, p-tau) in older adults (Jiang et al., 2016), amnestic mild cognitive impairment (aMCI) subjects (Canuet et al., 2015) and mild AD patients (Li et al., 2013; Celebi et al., 2016), no study has investigated whether CR is able to mitigate the effects of the pathology in network efficiency through graph theory analysis. Thus, in the present study we mainly sought to investigate the moderator effect of CR (if any) on the association between pathophysiology and functional network efficiency in aMCI and mild AD patients (i.e., can CR modify the effects of pathology on network topology?) as a primary outcome. As secondary outcomes, we analyzed the relationship between CR proxies and functional network efficiency (resting-state fMRI graph metrics) in healthy controls, aMCI and mild AD patients; the relationship between pathophysiology (CSF Aβ, total tau (t-tau) and p-tau levels) and functional network efficiency in aMCI and mild AD patients; and the relationship between pathophysiology and CR proxies in aMCI and mild AD patients.

## Materials and Methods

### Participants

This study included 70 participants: 14 mild AD patients, 28 aMCI subjects, and 28 healthy controls. To indicate dementia severity, we used ratings on the Clinical Dementia Rating (Morris, 1993) scale and a semi-structured interview about the patients’ functional level in their daily activities (Pfeffer et al., 1982). The diagnosis of probable AD patients fulfilled the standards of the National Institute of Aging and Alzheimer’s Association (NIA/AA; McKhann, 2011), and patients had a Clinical Dementia Rating score of 1. aMCI patients were diagnosed using the core criteria of the NIA/AA for MCI (Albert et al., 2011) and had a Clinical Dementia Rating score of 0.5 (with an obligatory memory score of 0.5). All aMCI subjects had memory complaints confirmed by a full range of neuropsychological testing, absence of dementia, and pathophysiological evidence of AD, characterized by low CSF Aβ_1–42_ (<416 pg/mL) and/or low Aβ_1–42_/p-tau (<9.53 pg/mL; Forlenza et al., 2015).

Controls were identified as cognitively normal: they did not exhibit any neurological or psychiatric disorders or require psychoactive medication; they demonstrated Mini Mental State Examination (MMSE) scores within the normal limits, considering scores corrected for educational level (Brucki et al., 2003); and their structural images were without any abnormalities. The control group had no memory complaints nor neurological deficits, and they also had a Clinical Dementia Rating score of zero. Exclusion criteria for all participants included: history of other neurological or psychiatric diseases or head injury with loss of consciousness, use of sedative drugs in the last 24 h before the neuropsychological assessment, drug or alcohol addiction, prior chronic exposure to neurotoxic substances, Hachinski ischemeic score (Hachinski et al., 1975) >4, and Fazekas Scale (Fazekas et al., 1987) >1.

Pre-diagnostic procedures also comprised laboratory tests including Vitamin B12, folate and thyroid hormones. The Medical Research Ethics Committee of University of Campinas (UNICAMP) approved this study and written informed consent (either from the subjects or from their responsible guardians, if incapable) was obtained from all participants before study initiation, according to the Declaration of Helsinki.

### Neuropsychological Assessment

Experienced neuropsychologists, blinded to the magnetic resonance imaging (MRI) data, performed the neuropsychological evaluations. These evaluations helped with the clinical assessment and diagnosis of patients. Detailed information for the neuropsychological testing can be found at Supplementary Materials.

### Cognitive Reserve

In the present study, we used years of education as a measure of CR (measured as the total number of years of schooling). Our study used a wide education range for all three groups, extending its low end to include no years of formal schooling (range of education in controls: 0–19 years; in aMCI: 0–23 years; in mild AD: 0–16 years). We did not dichotomize the subjects into high or low CR groups and treated the data as continuous variables in all analyses.

### Cerebrospinal Fluid Assessment

CSF samples from aMCI and AD patients were collected by lumbar puncture and stored in a polypropylene tube of 1 ml. Then the samples were centrifuged at 800 rpm for 10 min and stored at −80°C until analysis. Aβ_1–42_, t-tau protein and p-tau protein were measured using Luminex xMAP plataform (Inno-Bia Alzbio3 immunoassay reagents, Innogenetics, Ghent, Belgium).

### MRI Acquisition

All MR images were acquired on a 3.0 T MRI Philips Achieva^®^ scanner. The following acquisition protocol was applied to each subject: (a) sagittal high-resolution T1-weighted (isotropic voxels of 1 × 1 × 1 mm^3^, TR/TE = 7/3.2 ms, FOV = 240 × 240 mm, 180 slices); (b) functional acquisitions (EPI) with TR/TE = 2000/30 ms, FOV = 240 × 240, isotropic voxels set to 3 × 3 × 3 mm^3^, and no gap with a total scan time of 6 min, resulting in 180 full brain volumes with 40 axial slices each. All participants were instructed to keep their eyes closed, to relax, to move as little as possible, and to not fall asleep. The total scan time was 30 min.

### Image Processing and Statistical Analysis

We performed the resting-state functional connectivity preprocessing and analysis using an in-house toolbox (UF^2^C^1^; de Campos et al., 2016) that runs in the MATLAB platform (2014b, The MathWorks Inc., Natick, MA, USA) with SPM12^2^. We performed the T1-weighted image coregistration with the fMRI mean image, tissue segmentation, and normalization. We preprocessed the functional images based on volumes realignment, normalization (unified segmentation method—MNI152), and smoothing (6 × 6 × 6 mm^3^ FWHM). Additionally, we performed regressions for head motion, white matter, and CSF global signals and band-pass filtering (0.008–0.1 Hz). No subjects in the present study exceeded the mean framewise displacement threshold (0.5 mm), and there were no significant differences in head movement among the groups (analysis of variance, ANOVA, *p* = 0.187).

We used the UF^2^C toolbox to construct functional connectivity matrices (i.e., the input for graph analysis), based on 70 functional regions-of-interest (ROIs)—corresponding to the 12 resting-state networks established elsewhere (Shirer et al., 2012; regions available at http://findlab.stanford.edu/functional_ROIs.html (Supplementary Table S1). Functional connectivities of all ROI pairs were estimated as Pearson’s correlation coefficients and then transformed to Fisher’s Z estimates using Fisher’s r-to-z transformation. Due to local atrophy present in aMCI and mild AD patients, the local average of time-series per ROI could include signal derived from non-gray matter voxels, thereby introducing artificial differences between patients and controls. To overcome this issue, time-series were extracted only from voxels that were included in the subject’s gray matter mask; furthermore, UF^2^C correlates each single ROI voxel time series with the average ROI time series (gray matter-masked). The voxel was included (into the average) if its correlation value was within the average + standard deviation (SD) of all correlations between the ROI-masked voxels.

Because the arbitrary thresholding and binarization processes in graph theoretical analysis often lead to loss of information (Rubinov and Sporns, 2011), we chose weighted correlation matrices (in which the links contain information about the connection strength). Negative weights were considered zero. The following 10 graph metrics were obtained for each subject to estimate network efficiency: local betweenness centrality (*B*c) and local eigenvector centrality (*v*) as measures of centrality; global and local clustering coefficient (C*glob* and C*loc*, respectively) and global transitivity (T) as measures of segregation; global (λ) and local (λ(v)) characteristic path length, and global and local efficiency (E*glob* and E*loc*, respectively) as measures of integration. In addition to all these metrics, we also calculated small-worldness (σ), a global measure that combines path length and clustering coefficient. To give a brief idea, higher betweenness centrality, eigenvector centrality, clustering coefficient, transitivity, efficiency and small-worldness values are features of more efficient networks. On the other hand, lower values of characteristic path length represent more efficient networks.

To see the relationship between CSF values and graph metrics (secondary outcome), for each group separately (i.e., controlling for clinical dementia severity), we added CSF values as independent variables and graph metrics as dependent variables in the GraphVar toolbox (version 1.02^3^; Kruschwitz et al., 2015). We then did the same for education and graph metrics. In the next step, to test whether education modifies the relationship between CSF biomarker levels and the topological organization of the functional connectome (primary outcome), we used regression models in which we included an interaction term between education and the CSF biomarker levels. We then verified whether including this interaction term added significantly to the model.

For each graph parameter analyzed, we created a random distribution of the parameter value (5,000 permutations). We then employed a network-based statistic—NBS (Zalesky et al., 2010a), a process that tests whether a set of multiple pairwise connections forms ROI–ROI pairs that would be highly unlikely to occur randomly. Finally, as we used the same general linear models to test the relationships between CR, pathophysiological levels (CSF proteins) and network topology (graph metrics) for all three groups (MCI, AD and controls), we employed a Bonferroni correction to further account for the multiplicity of tests.

### Non-imaging Statistical Analysis

We first tested the normal distribution with Kolmogorov-Smirnov test for all non-imaging variables. The chi-square test was used for categorical variables comparison, such as sex. Differences between groups for demographic and years of education were tested with ANOVAs, Bonferroni *post hoc*. Group comparison for CSF variables was performed with *t*-tests (aMCI vs. mild AD group). Pearson’s correlation analyses were used to investigate the associations among demographic data (age and sex) with education. To investigate the relationship between CR and CSF biomarker levels in both aMCI and mild AD patients (secondary outcome), we used linear regression models, including the CSF data as dependent variables, and sex, age and education as independent variables. All statistical analyses were carried out using the *Statistical Package for the Social Sciences* (SPSS) program, version 22. The level of significance accepted was *α* = 0.05.

## Results

### Participants Characteristics

There was no difference in sex (*p* = 0.871) and educational level among the groups; and aMCI and mild AD groups did not statistically differ in CSF biomarker levels (Table 1). Controls were younger than AD patients (*p* = 0.009), and this variable was included as a confounding factor throughout the analysis. There was no statistically significant relationship between education and age or sex in controls, aMCI nor mild AD patients (data not shown). Neuropsychological performance results can be found at Supplementary Table S2.

**Table 1:** Group comparison for demographic and cerebrospinal fluid (CSF) proteins data.

	Controls	aMCI	Mild Alzheimer’s disease	Controls vs. aMCI	Controls vs. mild Alzheimer’s disease	aMCI vs. mild Alzheimer’s disease
N (female)	28 (19)	28 (17)	14 (9)			
Age	66.36 (5.6)	70.38 (6.9)	72.67 (6.8)	0.097	0.019	0.93
MMSE	28 (1.3)	25.34 (2.9)	19.8 (5.1)	0.013	<0.001	<0.001
Education	10.37 (5.2)	7.59 (5.5)	8.93 (5.2)	0.242	1	1
t-tau	NA	101.73 (66.1)	132.07 (76.2)	NA	NA	0.465
p-tau	NA	50.15 (32.3)	54.9 (32.2)	NA	NA	1
Aβ_1–42_	NA	428.83 (151.9)	355.89 (121.1)	NA	NA	0.316

*Notes: mean (sd). aMCI, amnestic mild cognitively impaired subjects; MMSE, Mini mental state examination; NA, not available*.

### Primary Outcome: The Interplay Between CR and Pathophysiology in Network Efficiency

**Figure 1:**
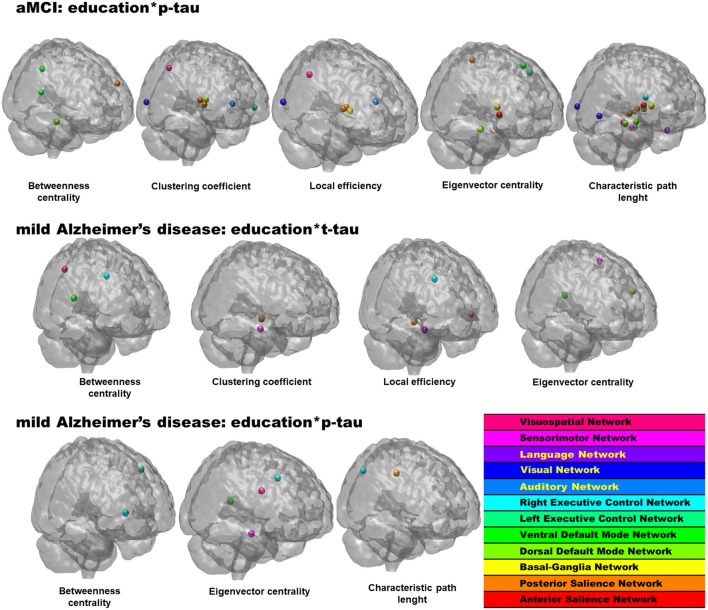
Statistically significant regions-of-interest (ROIs) for the interplay between educational level and pathophysiology in the network topology in amnestic mild cognitive impairment (aMCI) and mild Alzheimer’s disease (AD) groups. *p* values and anatomical brain regions in Table 2.

At this level, an interaction term between CSF proteins and education was added to the models, and when significant, the interaction term indicated an interplay between pathophysiological level and CR on network topology. As shown in Table 2, Figure 1 the interaction term between years of education and CSF p-tau level was a statistically significant predictor of graph metrics for the aMCI group. Similarly, interaction terms between education and both CSF t-tau and p-tau added significantly to models in the mild AD group, indicating that these interactions affect network topology. No results were observed for interaction terms involving CSF Aβ_1–42_.

**Table 2:** Statistically significant linear contents regression models examining interaction terms in network topology measures.

Interaction term	Graph metric	Anatomical region (network)	*p* value
**aMCI**			
Education*p-tau	Characteristic path length	NA	0.023
	Betweenness centrality	Left middle frontal gyrus (pSN)	0.003
		Right hippocampus (dDMN)	0.011
		Left middle occipital gyrus (vDMN)	0.006
		Right retrosplenial cortex, posterior cingulate cortex (vDMN)	0.004
	Clustering coefficient	Right thalamus (pSN)	0.008
		Left thalamus (pSN)	0.002
		Right brainstem/midbrain (BGN)	0.004
		Left inferior frontal gyrus, orbitofrontal gyrus (lECN)	0.011
		Left superior temporal gyrus (AN)	0.009
		Right middle occipital gyrus (VN)	0.019
		Right inferior parietal gyrus (VSN)	0.018
	Local efficiency	Right thalamus (pSN)	0.010
		Left thalamus (pSN)	0.004
		Right brainstem/midbrain (BGN)	0.001
		Left superior temporal gyrus (AN)	0.024
		Right middle occipital gyrus (VN)	0.005
		Right inferior parietal gyrus (VSN)	0.017
	Eigenvector centrality	Right insula (aSN)	0.002
		Left precuneus (pSN)	0.006
		Right brainstem/midbrain (BGN)	0.023
		Right hippocampus (dDMN)	<0.001
		Left middle frontal gyrus (vDMN)	0.010
		Left middle frontal gyrus, superior frontal gyrus (lECN)	<0.001
	Characteristic path length	Right insula (aSN)	0.022
		Right posterior insula (pSN)	0.008
		Left thalamus (pSN)	0.006
		Right brainstem/midbrain (BGN)	<0.001
		Left and right thalamus (dDMN)	0.016
		Left hippocampus (dDMN)	<0.001
		Right hippocampus (dDMN)	<0.001
		Left parahipocampal gyrus (vDMN)	0.011
		Left thalamus (lECN)	<0.001
		Right caudate (rECN)	0.002
		Left middle occipital gyrus (VN)	<0.001
		Right middle occipital gyrus (VN)	<0.001
		Left middle temporal gyrus (LN)	0.002
		Cerebellum (SMN)	0.004
		Left inferior temporal gyrus (VSN)	<0.001
**Mild Alzheimer’s disease**
Education*t-tau	Betweenness centrality	Right retrosplenial cortex, posterior cingulate cortex (vDMN)	0.004
		Right middle frontal gyrus, superior frontal gyrus (rECN)	0.002
		Right inferior parietal gyrus (VSN)	0.014
	Clustering coefficient	Right posterior insula (pSN)	0.007
		Cerebellum (SMN)	0.015
	Local efficiency	Left insula (aSN)	0.024
		Right posterior insula (pSN)	0.006
		Right middle frontal gyrus, superior frontal gyrus (rECN)	0.022
		Cerebellum (SMN)	0.013
	Eigenvector centrality	Left inferior frontal gyrus (BGN)	0.002
		Left retrosplenial cortex, posterior cingulate cortex (vDMN)	0.004
		Left precentral gyrus (SMN)	0.003
Education*p-tau	Betweenness centrality	Left middle frontal gyrus, superior frontal gyrus (lECN)	<0.001
		Right middle frontal gyrus (rECN)	0.001
	Eigenvector centrality	Left retrosplenial cortex, posterior cingulate cortex (vDMN)	0.013
		Right middle frontal gyrus, superior frontal gyrus (rECN)	0.002
		Cerebellum (SMN)	0.015
		Right frontal operculum, inferior frontal gyrus (VSN)	0.024
	Characteristic path length	Right middle cingulate cortex (pSN)	0.007
		Right inferior parietal gyrus, supramarginal gyrus, angular gyrus (rECN)	0.021

*Notes: aMCI, amnestic mild cognitively impaired subjects; CSF, cerebrospinal fluid; dDMN, dorsal Default Mode Network; vDMN, ventral Default Mode Network; lECN, left Executive Control Network; AN, Auditory Network; VSN, Visuospatial Network; aSN, anterior Salience Network; rECN, right Executive Control Network; SMN, Sensorimotor Network; pSN, posterior Salience Network; LN, Language Network; BGN, Basal Ganglia Network*.

### Secondary Outcomes

#### Group Comparison for Network Efficiency

Compared to controls, the aMCI group presented increased betweenness centrality (*B*c), local clustering coefficient (C*loc*), local efficiency (E*loc*) and eigenvector centrality (*v*) in several regions belonging to the Default Mode Network (DMN), Executive Control Network (ECN), Auditory Network (AN), Salience Network (SN), Visuospatial Network (VSN) and Sensorimotor Network (SMN); whereas diminished betweenness centrality (*B*c) and eigenvector centrality (*v*) were observed in regions belonging to the DMN, ECN and AN. Interestingly, although belonging to a variety of different networks, the regions that presented features of increased network efficiency in the aMCI were predominantly located in the frontal region. These findings suggest that predementia subjects present frontal nodes that work more efficiently than the cognitively healthy.

Compared to controls, mild AD patients presented increased local characteristic path length (λ(v)) in three regions belonging to the SN, DMN and language network (LN), indicating a possible disruption in these network’s information processing. aMCI subjects presented increased centrality values (*B*c and *v*) in regions of the DMN, SN, basal ganglia network (BGN) and VSN compared to mild AD patients; whereas decreased local characteristic path length (λ(v)) and centrality metrics (*B*c and *v*) were present in regions of the ECN, DMN, SMN and VSN (Supplementary Table S3).

#### Relationship Between Education and Pathophysiology

Multiple linear regression models failed to detect any statistically significant relationship between CSF biomarker levels and education in aMCI or mild AD groups, suggesting that the level of CR has no influence on brain AD-related pathology levels (Supplementary Table S4).

#### Relationship Between Education and Network Efficiency

In the control group, years of education had a positive correlation with local clustering coefficient (C*loc*) and local efficiency (E*loc*) in areas belonging to the SN, DMN, AN, VSN, LN and AN (the higher the education, the higher the C*loc* and E*loc* in these areas); whereas, there was negative correlation with local characteristic path length (λ(v)) in regions belonging to the DMN, AN, LN and VSN (the higher the education, the lower the λ(v)). In the aMCI group, the level of education had a positive relationship with the betweenness centrality (*B*c), local clustering coefficient (C*loc*) and local efficiency (E*loc*) of regions belonging to the SN, ECN, VSN and DMN. A negative relationship was observed between education and betweenness centrality (*B*c) at a region belonging to the VSN. In the mild AD group, years of education positively correlated with betweenness centrality (*B*c), local clustering coefficient (C*loc*), eigenvector centrality (*v*) and local efficiency (E*loc*) of regions belonging to the ECN, LN and DMN (Table 3). These findings indicate that healthy controls, aMCI and mild AD patients with higher level of CR present characteristics of more efficient networks.

**Table 3:** Statistically significant linear contents regression models examining relation of educational level and graph metrics in controls, aMCI and mild Alzheimer’s disease groups.

CR proxy	Graph metric	Anatomical region (network)	*p* value	β
**Controls**				
Education	Clustering coefficient	Right posterior insula (pSN)	0.016	**0.414**
		Left and right thalamus (dDMN)	0.002	**0.564**
		Left superior temporal gyrus (AN)	0.004	**0.469**
		Right inferior temporal gyrus (VSN)	0.011	**0.363**
	Local efficiency	Left and right thalamus (dDMN)	0.001	**0.580**
		Left superior temporal gyrus (AN)	0.001	**0.516**
		Left middle temporal gyrus, angular gyrus (LN)	0.010	**0.409**
		Right inferior temporal gyrus (VSN)	0.013	**0.363**
	Characteristic path length	Left and right thalamus (dDMN)	0.004	**−0.382**
		Left superior temporal gyrus (AN)	0.014	**−0.404**
		Left middle temporal gyrus, angular gyrus (LN)	0.007	**−0.448**
		Right inferior temporal gyrus (VSN)	0.005	**−0.354**
**aMCI**				
Education	Betweenness centrality	Anterior cingulate cortex, medial prefrontal cortex, supplementary motor area (aSN)	0.011	**0.433**
		Right inferior parietal gyrus, supramarginal gyrus, angular gyrus (rECN)	0.004	**0.481**
		Left inferior parietal gyrus (VSN)	0.007	−0.323
	Clustering coefficient	Right middle frontal gyrus (rECN)	0.014	**0.410**
	Local efficiency	Posterior cingulate cortex, precuneus (dDMN)	0.011	**0.467**
		Right middle frontal gyrus (rECN)	0.012	**0.442**
**Mild Alzheimer’s disease**
Education	Betweenness centrality	Left middle frontal gyrus, superior frontal gyrus (lECN)	<0.001	**0.675**
		Left superior parietal gyrus, inferior parietal gyrus, precuneus, angular gyrus (lECN)	0.001	**0.501**
		Left middle temporal gyrus (LN)	0.001	**0.728**
	Clustering coefficient	Medial prefrontal cortex, anterior cingulate cortex, orbitofrontal cortex (dDMN)	0.008	**0.685**
	Eigenvector centrality	Right superior frontal gyrus (dDMN)	0.001	**0.565**
	Local efficiency	Medial prefrontal cortex, anterior cingulate cortex, orbitofrontal cortex (dDMN)	0.002	**0.715**

*Notes: aMCI, amnestic mild cognitively impaired subjects; dDMN, dorsal Default Mode Network; vDMN, ventral Default Mode Network; lECN, left Executive Control Network; AN, Auditory Network; VSN, Visuospatial Network; aSN, anterior Salience Network; rECN, right Executive Control Network; SMN, Sensorimotor Network; pSN, posterior Salience Network; LN, Language Network; BGN, Basal Ganglia Network; β, linear regression coefficient. In bold, expected direction for the relationship*.

#### Relationship Between Pathophysiology and Network Efficiency

Before hand, we think it is helpful to describe the expected concentration of t-tau, p-tau and Aβ_1–42_ extracted from the CSF of patients. Because molecules of Aβ_1–42_ are composing amyloid plaques in the brain, low quantities diffuse to the CSF in AD (Blennow and Zetterberg, 2009), in which CSF Aβ_1–42_ correlates inversely with total Aβ load in the brain (Tapiola et al., 2009). In contrast, high levels of CSF t-tau and p-tau are correlated with the presence of neocortical neurofibrillary tangles (Tapiola et al., 2009). Hence, AD patients are expected to present lower levels of CSF Aβ_1–42_, but higher levels of both CSF t-tau and p-tau than cognitively healthy elderlies.

Overall, the relationship between CSF biomarkers and graph metrics did not follow an expected pattern for the aMCI group, in which both positive and negative correlations were observed for t-tau, p-tau and Aβ_1–42_ levels. Interestingly, the relationship between CSF protein levels and graph metrics in the mild AD group was mostly unexpected: with few exceptions, the brain pathological features (high levels of t-tau and p-tau, and low levels of Aβ_1–42_) were related to features of more efficient networks. For example, higher levels of both t-tau and p-tau were related to increased betweenness (*B*c), clustering coefficient (C*loc*), local efficiency (E*loc*) and eigenvector centrality (*v*) and decreased characteristic path length (λ(v)) in several regions. Likewise, lower levels of Aβ_1–42_ were related to increased betweenness (*B*c) and eigenvector centrality (*v*) in some regions belonging to the SMN and VSN (Table 4).

**Table 4:** Statistically significant linear contents regression models examining the relationship of CSF biomarkers and graph metrics in aMCI and mild Alzheimer’s disease groups.

CSF protein	Graph metric	Anatomical region (network)	*p* value	β
**aMCI**				
t-tau	Betweenness centrality	Right inferior frontal gyrus (BGN)	0.023	**−0.460**
	Eigenvector centrality	Right brainstem/midbrain (BGN)	0.008	0.407
		Right frontal operculum, inferior frontal gyrus (VSN)	0.006	0.493
p-tau	Betweenness centrality	Left superior parietal gyrus, inferior parietal gyrus, precuneus, angular gyrus (lECN)	0.015	0.519
		Left middle temporal gyrus, angular gyrus (LN)	0.022	0.615
	Local efficiency	Left superior temporal gyrus (AN)	0.013	**−0.387**
	Eigenvector centrality	Left parahipocampal gyrus (vDMN)	0.014	**−0.413**
		Right angular gyrus, middle occipital gyrus (vDMN)	0.023	0.483
		Right middle frontal gyrus, superior frontal gyrus (rECN)	0.002	0.443
		Right frontal operculum, inferior frontal gyrus (VSN)	0.011	0.459
	Characteristic path length	Left superior temporal gyrus (AN)	0.016	**0.510**
Aβ_1–42_	Betweenness centrality	Left thalamus (lECN)	0.021	**0.406**
		Right frontal operculum, inferior frontal gyrus (VSN)	0.009	**0.340**
	Clustering coefficient	Right angular gyrus (dDMN)	0.008	−0.511
	Local efficiency	Posterior cingulate cortex, precuneus (dDMN)	0.021	−0.428
		Right angular gyrus (dDMN)	0.008	−0.513
	Eigenvector centrality	Left precuneus (pSN)	0.005	−0.480
		Left inferior frontal gyrus (BGN)	0.009	**0.514**
		Right superior frontal gyrus, middle frontal gyrus (vDMN)	0.020	**0.431**
		Right supplementary motor area (SMN)	0.020	−0.388
**Mild Alzheimer’s disease**				
t-tau	Global efficiency	NA	0.024	0.605
	Smallworldness	NA	0.005	−0.697
	Betweenness centrality	Posterior cingulate cortex, precuneus (dDMN)	0.008	0.582
		Right middle frontal gyrus (rECN)	0.004	0.609
		Left inferior parietal gyrus (VSN)	0.002	**−0.593**
		Right frontal operculum, inferior frontal gyrus (VSN)	0.019	**−0.513**
	Clustering coefficient	Left precuneus (pSN)	0.006	0.535
		Left hippocampus (dDMN)	0.017	0.719
		Right hippocampus (dDMN)	0.008	0.626
		Left parahipocampal gyrus (vDMN)	0.009	0.647
		Right superior frontal gyrus, middle frontal gyrus (vDMN)	0.004	0.579
		Right middle frontal gyrus, superior frontal gyrus (rECN)	0.013	0.500
		Right superior frontal gyrus (rECN)	0.001	0.601
		Right caudate (rECN)	0.020	0.545
		Left middle frontal gyrus, superior frontal gyrus, precentral gyrus (VSN)	0.007	0.545
		Left frontal operculum, inferior frontal gyrus (VSN)	0.022	0.522
		Right frontal operculum, inferior frontal gyrus (VSN)	0.013	0.555
	Local efficiency	Left precuneus (pSN)	0.011	0.500
		Right brainstem/midbrain (BGN)	0.020	0.582
		Midcingulate cortex (dDMN)	0.204	0.350
		Right hippocampus (dDMN)	0.006	0.583
		Left parahipocampal gyrus (vDMN)	0.008	0.622
		Right superior frontal gyrus, middle frontal gyrus (vDMN)	0.003	0.593
		Right superior frontal gyrus (rECN)	0.001	0.592
		Right caudate (rECN)	0.020	0.535
		Left middle frontal gyrus, superior frontal gyrus, precentral gyrus (VSN)	0.017	0.524
		Left frontal operculum, inferior frontal gyrus (VSN)	0.019	0.529
	Eigenvector centrality	Left precuneus (pSN)	<0.001	0.735
		Right middle frontal gyrus, superior frontal gyrus (rECN)	0.001	0.670
		Right precentral gyrus (SMN)	0.010	0.589
	Characteristic path length	Right middle frontal gyrus (aSN)	0.015	−0.657
		Left precuneus (pSN)	0.018	−0.590
		Right superior frontal gyrus (dDMN)	0.019	−0.642
		Left and right thalamus (dDMN)	0.010	−0.613
		Right superior frontal gyrus (rECN)	<0.001	−0.719
		Left middle occipital gyrus (VN)	0.003	−0.449
		Right middle occipital gyrus (VN)	0.015	−0.482
p-tau	Betweenness centrality	Posterior cingulate cortex, precuneus (dDMN)	0.011	0.574
		Left hippocampus (dDMN)	0.003	0.695
		Left inferior parietal gyrus (VSN)	0.001	**−0.611**
		Right frontal operculum, inferior frontal gyrus (VSN)	0.019	**−0.527**
	Clustering coefficient	Left precuneus (pSN)	0.012	0.520
		Posterior cingulate cortex, precuneus (dDMN)	0.007	0.610
		Right caudate (rECN)	0.017	0.572
	Local efficiency	Left precuneus (pSN)	0.006	0.532
		Posterior cingulate cortex, precuneus (dDMN)	0.002	0.634
		Right hippocampus (dDMN)	0.010	0.563
		Right caudate (rECN)	0.016	0.535
		Right inferior parietal gyrus (VSN)	0.022	0.501
	Eigenvector centrality	Right middle frontal gyrus, superior frontal gyrus (rECN)	0.011	0.562
	characteristic path length	Left precuneus (pSN)	<0.001	−0.768
		Posterior cingulate cortex, precuneus (dDMN)	0.018	−0.662
		Left and right thalamus (dDMN)	0.005	−0.655
		Left hippocampus (dDMN)	0.007	−0.509
		Right hippocampus (dDMN)	0.003	−0.580
		Precuneus (vDMN)	0.022	−0.671
		Left middle occipital gyrus (VN)	0.007	−0.412
		Right middle temporal gyrus, superior temporal gyrus, supramarginal gyrus, angular gyrus (LN)	0.020	−0.506
		Right inferior parietal gyrus (VSN)	0.023	−0.717
		Right inferior temporal gyrus (VSN)	0.007	**0.530**
Aβ_1–42_	Betweenness centrality	Left precentral gyrus (SMN)	0.016	−0.603
	Eigenvector centrality	Left inferior temporal gyrus (VSN)	0.017	−0.595
		Right middle frontal gyrus (VSN)	0.009	**0.527**

*Notes: aMCI, amnestic mild cognitively impaired subjects; CSF, cerebrospinal fluid; dDMN, dorsal Default Mode Network; vDMN, ventral Default Mode Network, lECN, left Executive Control Network; AN, Auditory Network; VSN, Visuospatial Network; aSN, anterior Salience Network; rECN, right Executive Control Network; SMN, Sensorimotor Network; pSN, posterior Salience Network; LN, Language Network; BGN, Basal Ganglia Network; β, linear regression coefficient. In bold, expected direction for the relationship*.

## Discussion

Previous studies have suggested that CR could explain why nearly one third of the cognitively healthy population met criteria for AD at necropsies without clinically expressing it (Bennett et al., 2006; Morris et al., 2010). In this context, we aimed to analyze the relationship between a CR proxy, CSF biomarker levels (pathology burden) and graph metrics (network topology), as well as whether CR could modify the relationship between pathology burden and network topology (i.e., the CR hypothesis). According to our findings, although higher levels of CR did not seem to protect individuals from developing the pathophysiological features of AD, cognitively healthy controls, aMCI and mild AD patients with higher level of CR presented features of more efficient networks. Altered pathological levels of CSF protein biomarkers were related to a dual pattern of network efficiency in aMCI, whereas they were related to more efficient networks in the mild AD group. We suppose that educational level could modify the effects of p-tau in network topology for the aMCI group, and the effects of t-tau and p-tau in network topology for the mild AD group. In what follows, we will discuss these points in turn.

### The Interplay Between CR and Pathophysiology in Network Efficiency

The analysis undertaken in the present work demonstrated that educational level—a proxy for CR—, has modifying effects on the relationship between evidence of AD pathophysiology and network topology in both aMCI and mild AD groups. While CR did not seem to protect individuals from developing the pathophysiological features of AD, it did seem to modify the association between pathology and resting-state network topology for both groups. A previous study claimed that CR can only compensate for pathology up to an initial phase and does not act in a certain stage of dementia (Serra et al., 2017). To test this hypothesis, however, the authors analyzed differences in graph metrics between high/low CR aMCI and mild AD patients and could only find differences for the aMCI subjects. In the present work, we have directly tested whether CR has any modifying effect in network topology, and we extend previous findings claiming that CR acts as a modifying factor even in the dementia phase. The relationship between higher burden of abnormal proteins and graph metrics for AD has been interpreted previously in the light of a compensatory mechanism. We could extend these results hypothesizing that at the dementia stage, this compensatory mechanism (i.e., enhanced network efficiency) is further increased by a high CR level. The findings with interaction terms give supplementary evidence for that, pointing that educational level can, indeed, modify the effect of pathology on network topology.

Individuals with higher educational level might use brain networks more efficiently. The biological underpinnings behind this association are yet to be further elucidated. *In vivo* studies have shown that animals living in an enriched environment restored neurogenesis (Ihunwo et al., 2016) and increased the number of surviving newborn progenitor-derived cells in the hippocampal dentate gyrus (Nilsson et al., 1999). The human brain also retains its ability to generate neurons throughout life (Johansson et al., 1999) and engagement in more cognitively stimulating activities might increase neurogenesis, synaptogenesis, levels of brain derived neurotrophic factor and related neurotrophins (van Praag et al., 2000), as well as change astroglial morphology and volume (Beauquis et al., 2013). CR may also involve upregulation of the noradrenergic system—which is depleted with age and AD (Robertson, 2013).

### Group Comparison for Network Efficiency

In the present study, we found that mild AD patients presented longer characteristic path length (λ(v)) than healthy controls, aligning with previous research (Stam et al., 2007; Sanz-Arigita et al., 2010; Supplementary Table S3). Some other studies have also found a decrease in the eigenvector centrality (*v*; Binnewijzend et al., 2014) and clustering coefficient (C*loc*; Supekar et al., 2008), and a diminished number of hubs (Khazaee et al., 2017) in patients, suggesting a randomization of the brain networks and a loss of information flow and integration. In the present work, the regions that presented longer characteristic path length (λ(v)) in mild AD (the right middle cingulate cortex, the left hippocampus and the left middle temporal gyrus) are the ones more commonly hit by the pathophysiological alterations, markedly presenting metabolic dysfunctions and atrophy. Thus, it is not surprising that these regions presented longer local path lengths (λ(v)) and lower capacity to combine information from their neighbors.

Studies involving aMCI subjects, in turn, are much less frequent. Some authors have reported a loss in “small-worldness” features in the aMCI group, such as an increased path length (λ(v); Wang et al., 2013) and decreased eigenvector centrality (*v*; Qiu et al., 2016) when compared to controls. Our aMCI subjects presented diminished measures of centrality in some regions, whereas other regions showed increased measurements of centrality, segregation and integration when compared to controls (Supplementary Table S3). Although unexpected, previous results (Qiu et al., 2016) can shed some light onto this issue regarding increases in “small-worldness” properties predominantly in frontal regions in our predementia subjects. The authors performed a longitudinal analysis in a MCI group and, interestingly, the ones that converted to dementia presented increased eigenvector centrality (*v*) in frontal regions when compared to the stable ones. The authors interpreted that the frontal regions became more important facing the alterations within the hippocampal network, with increased eigenvector centrality in those regions consisting of a mechanism of functional compensation.

### Relationship Between Education and Pathophysiology

Consistent to previous studies (Brayne et al., 2010), we did not find any associations between pathophysiological measurements and CR proxies (Supplementary Table S4). Our results suggest that educational level does not protect individuals from developing pathology. The divergence between our results and others could be explained by other factors associated to higher CR proxies, which could act as confounding factors (Del Ser et al., 1999). Additionally, cognitively stimulating activities could play different roles during pre- and post-amyloid plaque stages, diminishing Aβ_1–42_ production in pre-amyloid plaque stages (Jagust and Mormino, 2011).

### Relationship Between Education and Network Efficiency

Converging evidence suggests that healthy individuals with higher CR proxies present greater volume and metabolism in some brain regions (Arenaza-Urquijo et al., 2013a; Rzezak et al., 2015). The same relationship is true for resting-state fMRI (Song et al., 2008) and graph analysis studies (van den Heuvel et al., 2009; Fischer et al., 2014; Santarnecchi et al., 2015), suggesting that healthy individuals with higher CR proxies present a greater processing capacity and network efficiency. Our results align with these previous findings: healthy controls with higher level of education presented higher clustering coefficient (C*loc*) and local efficiency (E*loc*) in areas belonging to the posterior Salience Network (pSN), dDMN, AN, VSN, LN and AN and lower characteristic path length (λ(v)) in regions belonging to the dDMN, AN, LN and VSN (Table 3).

By contrast, the interpretation of the protective effects of CR in AD patients could be tricky. For example, structural MRI studies have consistently found that MCI and AD patients with higher CR proxies present reduced brain volumes and/or thickness (Solé-Padullés et al., 2009; Arenaza-Urquijo et al., 2013b), suggesting that they can tolerate a more advanced neurodegenerative process when a certain clinical condition is reached. In fMRI studies, though, patients with higher CR present higher brain activation during task performance (Bosch et al., 2010; Colangeli et al., 2016), and higher DMN functional connectivity (Bozzali et al., 2015) and network efficiency (Franzmeier et al., 2017) during resting-state. Consistent with these previous results, we found that both aMCI and mild AD patients with higher levels of education generally presented graph metrics suggestive of more efficient network topologies in regions belonging to several networks. These findings will be discussed in the light of two complementary facets to the neural implementation of the CR hypothesis: neural reserve and neural compensation (Stern, 2002).

Neural reserve may represent innate occurring individual differences in brain networks that may be modulated through life events (Stern et al., 2005). In this context, the positive relationship between efficiency of networks and educational level in both aMCI and mild AD patients may be explained by the pre-existing high levels of network efficiency before the development of the disease. Alternatively, it could reflect a compensatory increase in the efficiency of networks facing the development of pathological features (i.e., neural compensation; Stern et al., 2005). Given that healthy individuals with higher CR proxies also presented higher network efficiency (i.e., the beneficial effects of education were observed in the absence of the disease), it is tempting to say that our results point towards the neural reserve explanation. However, we did not obtain pathophysiological measurements for healthy controls, and it is known that the pathological burden starts even decades before the onset of dementia, and cognitively healthy subjects could present amyloidosis even without cognitive symptoms. Thus, we cannot discard the possibility that some examples of our healthy sample presented AD pathophysiological features, and it could be that a “neural compensation-like” mechanism is already taking place in some cognitively normal subjects.

The present work brings evidence of an association between educational level and network efficiency not only in the healthy elderly, but also among the aMCI and the demented. It is yet to be found, however, whether possessing higher CR proxies promotes network efficiency or that those subjects with more efficient networks tend to engage more in stimulating activities (Scarmeas and Stern, 2003).

### Relationship Between Pathophysiology and Network Efficiency

Overall, we found that aMCI CSF biomarker levels were associated with a dual pattern of network topology (Table 4), similarly to previous research reporting that CSF markers of amyloid deposition and neuronal injury in MCI were associated with both increased and decreased functional connectivity of resting-state networks (Canuet et al., 2015). In the mild AD sample, in turn, we could observe some more notorious results: generally, pathological levels of CSF proteins were related to features of more efficient networks (mostly, but not limited to, the DMN). Divergent results from our and previous work (such as in Binnewijzend et al., 2014), in which the authors report no significant correlations of eigenvector centrality (*v*) with CSF biomarker) may have arisen from different methodologies adopted.

Since the pathophysiological process is known to start during a preclinical phase, protein levels have been related to alterations in functional connectivity (Wang L. et al., 2013) and graph metrics (Brier et al., 2014) in cognitively normal older individuals with evidence of preclinical AD. At a further stage, such as in MCI subjects for example, there could be an inconsistent adaptation process arising as a result of higher protein abnormalities and structural brain damage. Such speculation could possibly explain the lack of relationship pattern between CSF protein levels and graph metrics found here. Mild AD patients, in turn, might have already reached a plateau for protein biomarkers (Jack et al., 2010) when a process of compensation takes place, explaining the relationship between higher pathological levels of CSF proteins and network efficiency. However, such interpretation is speculative and longitudinal studies would be necessary to confirm it.

## Limitations and Conclusion

This work has some limitations that must be acknowledged. First, its relative small sample size and the lack of CSF data for the control sample. Second, its cross-sectional nature does not allow for understanding the effect CR may have on neuropathological accumulation and network topology over time. Third, it is possible that the relationship between CR proxies and network topology, as well as the modifying effects of education are not directly or linearly related to CR. For instance, higher educational level is usually related to higher levels of indexes of socioeconomic status and lifestyle that have been shown to mitigate the risk of AD (Lu et al., 2016). Relatedly, it has been shown that being engaged in intellectual leisure, social and physical activities reduces the risk of developing dementia (Scarmeas et al., 2001). In the present study, we did not evaluate personal engagement in such activities, nor cardiovascular risk factors or unhealthy lifestyles. Brain volume/size was also claimed to protect against clinical deterioration (Guo et al., 2013), and this variable was not accounted for.

We should not forget to mention the contribution of some confounding factors to the graph analysis approach as well. Whereas most studies have used the AAL template for defining nodes, we chose our nodes based on a functional atlas of resting-state networks; and it has been demonstrated that both local and global topological properties of networks exhibit strong dependence on the choice of parcellation scale (Zalesky et al., 2010b). Furthermore, some studies have used thresholds to produce binary adjacency matrices, which generate graphs of different sparsity or connection density (Bullmore and Sporns, 2009). Lastly, unanalyzed biological factors such as the presence of *APOE*4 gene have been associated with disrupted graph topologies (Daianu et al., 2015; Wang et al., 2015).

Over the last few decades, many authors have committed to studying the relationships and effects of pursuing higher CR levels for the brain. Such studies are of utmost importance because not only do they provide evidence of the beneficial effects of being immersed in a cognitively stimulating environment, but also highlight that interventions might be useful for slow age-related cognitive decline or dementia and to lengthen healthy aging. The current study has the strength of combining a wide range of educational levels that are not often seen in European or North-American studies, and could potentially lead to new insights into the mechanisms of CR. In summary, our findings suggest that although higher levels of CR did not seem to protect individuals from developing the pathophysiological features of AD, cognitively healthy controls, aMCI and demented patients with higher level of CR presented features of more efficient networks. Moreover, educational level could modify the effects of p-tau in network topology in the aMCI group, and the effects of t-tau and p-tau in network topology in the mild AD group; meaning that subjects with higher CR are better able to cope with the effects of pathology in terms of network efficiency.

## Author Contributions

MW: conception and design, data collection, data analysis and interpretation, article drafting and critical revision, final approval of version to be published. RFC and MLFB: conception and design, data analysis and interpretation, article drafting and critical revision, final approval of version to be published. BMC: data analysis and interpretation, article drafting, final approval of version to be published. CVLT, AFMKC-C, JEV, TNCM, DQA, LLT and OVF: data collection, article drafting, final approval of version to be published. GC: conception and design of the work, data analysis and interpretation, article drafting and critical revision, final approval of version to be published.

## Conflict of Interest Statement

The authors declare that the research was conducted in the absence of any commercial or financial relationships that could be construed as a potential conflict of interest. The reviewer CS declared a shared affiliation, though no other collaboration, with several of the authors LLT and OVF to the handling Editor at the time of the review.

## Footnotes

^1^https://www.lniunicamp.com/uf2c

^2^http://www.fil.ion.ucl.ac.uk/spm/

^3^http://www.nitrc.org/projects/graphvar/
