# Supplementary material for: Cognitive Reserve Relates to Functional Network Efficiency in Alzheimer’s Disease
Source: Front Aging Neurosci. 2018 Aug 21;10:255. doi: 10.3389/fnagi.2018.00255 (PMC6111617; doi:10.3389/fnagi.2018.00255)
Supplement: Supplementary file 1 [file Table_1.docx]

**Table S1**: Anatomical location of the seventy regions of interest (corresponding to twelve resting-state networks) used to characterize the topological organization of the human connectome with graph metrics (ROIs obtained from <http://findlab.stanford.edu/functional_ROIs.html>).

| **Network** | **Anatomical location** | **ROI size (voxels)** |
| --- | --- | --- |
| Anterior Salience | Left middle frontal gyrus | 651 |
|  | Left insula | 305 |
|  | Anterior cingulate cortex, medial prefrontal cortex, supplementary motor area | 2887 |
|  | Right middle frontal gyrus | 470 |
|  | Right insula | 319 |
| Posterior Salience | Left middle frontal gyrus | 93 |
|  | Left supramarginal gyrus, inferior parietal gyrus | 1205 |
|  | Left precuneus | 98 |
|  | Right middle cingulate cortex | 56 |
|  | Right precuneus, superior parietal gyrus | 133 |
|  | Right supramarginal gyrus, inferior parietal gyrus | 1002 |
|  | Left thalamus | 142 |
|  | Left posterior insula | 114 |
|  | Right thalamus | 63 |
|  | Right posterior insula | 134 |
| Dorsal Default Mode | Medial prefrontal cortex, anterior cingulate cortex, orbitofrontal cortex | 5257 |
|  | Left angular gyrus | 97 |
|  | Right superior frontal gyrus | 137 |
|  | Posterior cingulate cortex, precuneus | 1555 |
|  | Midcingulate cortex | 114 |
|  | Right angular gyrus | 38 |
|  | Left and right thalamus | 220 |
|  | Left hippocampus | 393 |
|  | Right hippocampus | 142 |
| Ventral Default Mode | Left retrosplenial cortex, posterior cingulate cortex | 462 |
|  | Left middle frontal gyrus | 405 |
|  | Left parahipocampal gyrus | 134 |
|  | Left middle occipital gyrus | 491 |
|  | Right retrosplenial cortex, posterior cingulate cortex | 590 |
|  | Precuneus | 1921 |
|  | Right superior frontal gyrus, middle frontal gyrus | 399 |
|  | Right parahipocampal gyrus | 90 |
|  | Right angular gyrus, middle occipital gyrus | 752 |
| Left Executive Control | Left middle frontal gyrus, superior frontal gyrus | 1501 |
|  | Left inferior frontal gyrus, orbitofrontal gyrus | 437 |
|  | Left superior parietal gyrus, inferior parietal gyrus, precuneus, angular gyrus | 2110 |
|  | Left inferior temporal gyrus, middle temporal gyrus | 350 |
|  | Left thalamus | 8 |
| Right Executive Control | Right middle frontal gyrus, superior frontal gyrus | 2093 |
|  | Right middle frontal gyrus | 356 |
|  | Right inferior parietal gyrus, supramarginal gyrus, angular gyrus | 1873 |
|  | Right superior frontal gyrus | 83 |
|  | Right caudate | 188 |
| Language | Left inferior frontal gyrus | 652 |
|  | Left middle temporal gyrus | 27 |
|  | Left middle temporal gyrus, angular gyrus | 317 |
|  | Left middle temporal gyrus, supramarginal gyrus, angular gyrus | 1420 |
|  | Right inferior frontal gyrus | 58 |
|  | Right middle temporal gyrus, supramarginal gyrus, angular gyrus | 1106 |
| Visuospatial  /Dorsal Attention | Left middle frontal gyrus, superior frontal gyrus, precentral gyrus | 338 |
|  | Left inferior parietal gyrus | 2020 |
|  | Left frontal operculum, inferior frontal gyrus | 1105 |
|  | Left inferior temporal gyrus | 93 |
|  | Right middle frontal gyrus | 97 |
|  | Right inferior parietal gyrus | 1193 |
|  | Right frontal operculum, inferior frontal gyrus | 326 |
|  | Right inferior temporal gyrus | 76 |
| Basal Ganglia | Right brainstem/midbrain | 669 |
|  | Left brainstem/midbrain | 828 |
|  | Left inferior frontal gyrus | 18 |
|  | Right inferior frontal gyrus | 63 |
| Auditory | Left superior temporal gyrus | 962 |
|  | Right superior temporal gyrus | 554 |
| Visual | Left middle occipital gyrus | 868 |
|  | Right middle occipital gyrus | 1679 |
|  | Cuneus/left calcarine | 1116 |
| Sensorimotor | Left precentral gyrus | 1365 |
|  | Right precentral gyrus | 1446 |
|  | Right supplementary motor area | 159 |
|  | Cerebellum | 2015 |
|  |  |  |

**Neuropsychological assessment**

Neuropsychologists administered to all participants a cognitive, functional and neuropsychiatric battery without knowledge of clinical diagnosis. Global cognitive status was measured by MMSE (Folstein, Folstein, & McHugh, 1975); episodic memory by the Rey Auditory Verbal Learning Test (subitems encoding, delayed recall, and recognition) (Malloy-Diniz, Lasmar, Gazinelli Lde, Fuentes, & Salgado, 2007); attention/working memory by forward digit span and backward digit span; for visual perception, we used the following tests: subtests of Luria’s Neuropsychological Investigation (Christensen, 1975), using items G12, G13, G14 and G17 (from Raven’s test), one item for mental rotation of figures (Ratcliff, 1979), and a copy of the Rey-Osterrieth Complex Figure Test (Osterrieth, 1944). For executive functions we used the Trail Making Test A and B (Christensen & Guilford 1959), the Stroop test (Stroop, 1935) and the Clock Drawing Test (Sunderland *et al*., 1989). Language tests included the Boston Naming Test (Kaplan, Goodglass, & Weintraub, 1983), semantic verbal fluency for category (animals), and phonological verbal fluency for letters. (Christensen & Guilford 1959).

**Table S2**: Group comparison for neuropsychological data.

|  | **controls** | **aMCI** | **mild Alzheimer’s disease** | **controls *vs* aMCI** | **controls *vs***  **mild Alzheimer’s disease** | **aMCI**  ***vs***  **mild Alzheimer’s disease** |
| --- | --- | --- | --- | --- | --- | --- |
| **Encoding** | 42.82(6.5) | 30.31(6.5) | 24.93(14.4) | **<0.001** | **<0.001** | 0.242 |
| **Delayed recall** | 8.89(2.0) | 4.25(1.8) | 1.33(1.5) | **<0.001** | **<0.001** | **<0.001** |
| **Recognition** | 12.37(2.5) | 4.59(5.8) | -0.27(5.59) | **<0.001** | **<0.001** | **0.014** |
| **Forward digit span** | 4.68(1.1) | 4.28(0.7) | 4.53(0.9) | 0.477 | 1 | 1 |
| **Backward digit span** | 4.21(0.8) | 3.13(0.8) | 3.47(0.9) | **<0.001** | 0.06 | 0.806 |
| **Stroop C time** | 35.78(7.4) | 44.75(15.3) | 66.87(48.2) | 0.636 | **<0.001** | **0.029** |
| **Stroop C errors** | 0(0) | 0.09(0.3) | 0.53(0.6) | 1 | **<0.001** | **0.029** |
| **Stroop I time** | 111.04(26.4) | 157.81(49.8) | 103.07(94.2) | **0.01** | 1 | **0.01** |
| **Stroop I errors** | 2.04(2) | 7.52(7.9) | 10.33(8.9) | **0.016** | **0.007** | 0.785 |
| **Semantic fluency** | 18.25(6.9) | 13.63(4) | 11(4.8) | **0.013** | **<0.001** | 0.553 |
| **Phonological fluency** | 33.89(10.5) | 24.53(9.8) | 20.13(13.1) | **0.011** | **0.004** | 0.760 |
| **Luria’s investigation** | 17.25(2.3) | 16.6(2.4) | 14.7(3.1) | 1 | **0.019** | 0.103 |
| **Clock drawing** | 8.93(1.2) | 7.26(1.9) | 5.8(2.7) | **0.011** | **<0.001** | 0.096 |
| **Rey copy** | 29.8(6.6) | 24.27(7.4) | 17.93(12.9) | 0.081 | **<0.001** | 0.103 |
| **Trail Making Test A** | 60.48(14.7) | 90.88(49.4) | 146.67(125.4) | 0.296 | **<0.001** | 1 |
| **Trail Making Test B** | 128.52(73.7) | 176.7(74.5) | 99(64.5) | 0.068 | 1 | 1 |
| **Boston Naming Test** | 56(4.9) | 51.16(10.5) | 44.93(25.9) | 0.691 | 1 | 1 |

Notes: mean (sd). aMCI: amnestic mild cognitively impaired subjects; NA: not available.

**Table S3:** Statistically significant graph metrics differences among controls, aMCI and mild Alzheimer’s disease patients.

| **Groups** | **Graph metric** | **Anatomical region (network)** | ***p*** | **direction** |
| --- | --- | --- | --- | --- |
| **controls *vs* aMCI** | betweenness centrality | Posterior cingulate cortex, precuneus (dDMN) | 0.007 | aMCI>controls |
|  |  | Right superior frontal gyrus, middle frontal gyrus (vDMN) | 0.007 | controls>aMCI |
|  |  | Left superior parietal gyrus, inferior parietal gyrus, precuneus, angular gyrus (lECN) | 0.014 | controls>aMCI |
|  |  | Left superior temporal gyrus (AN) | 0.038 | aMCI>controls |
|  |  | Left middle frontal gyrus, superior frontal gyrus, precentral gyrus (VSN) | 0.037 | aMCI>controls |
|  | eigenvector centrality | Anterior cingulate cortex, medial prefrontal cortex, supplementary motor area (aSN) | 0.046 | aMCI>controls |
|  |  | Right inferior parietal gyrus, supramarginal gyrus, angular gyrus (rECN) | 0.034 | controls>aMCI |
|  |  | Right superior temporal gyrus (AN) | 0.006 | controls>aMCI |
|  |  | Left precentral gyrus (SMN) | 0.017 | aMCI>controls |
|  | clustering coefficient | Left inferior frontal gyrus, orbitofrontal gyrus (lECN) | 0.030 | aMCI>controls |
|  | local efficiency | Left angular gyrus (dDMN) | 0.049 | aMCI>controls |
|  |  | Left and right thalamus (dDMN) | 0.043 | aMCI>controls |
|  |  | Left inferior frontal gyrus, orbitofrontal gyrus (lECN) | 0.025 | aMCI>controls |
| **controls *vs* Alzheimer’s** | characteristic path length | Right middle cingulate cortex (pSN) | 0.044 | Alzheimer’s>controls |
|  |  | Left hippocampus (dDMN) | 0.024 | Alzheimer’s>controls |
|  |  | Left middle temporal gyrus, angular gyrus (LN) | 0.036 | Alzheimer’s>controls |
| **aMCI *vs* Alzheimer’s** | betweenness centrality | Precuneus (vDMN) | 0.025 | aMCI>Alzheimer’s |
|  |  | Right middle frontal gyrus (rECN) | 0.046 | Alzheimer’s>aMCI |
|  | eigenvector centrality | Left supramarginal gyrus, inferior parietal gyrus (pSN) | 0.013 | aMCI>Alzheimer’s |
|  |  | Right brainstem/midbrain (BGN) | 0.042 | aMCI>Alzheimer’s |
|  |  | Precuneus (vDMN) | 0.014 | Alzheimer’s>aMCI |
|  |  | Left precentral gyrus (SMN) | 0.034 | Alzheimer’s>aMCI |
|  |  | Left middle frontal gyrus, superior frontal gyrus, precentral gyrus (VSN) | 0.026 | aMCI>Alzheimer’s |
|  | characteristic path length | Right inferior temporal gyrus (VSN) | 0.009 | Alzheimer’s>aMCI |

Notes: aMCI: amnestic mild cognitively impaired subjects; dDMN: dorsal Default Mode Network; vDMN: ventral Default Mode Network; lECN: left Executive Control Network; AN: Auditory Network; VSN: Visuospatial Network; aSN: anterior Salience Network; rECN: right Executive Control Network; SMN: Sensorimotor Network; pSN: posterior Salience Network; LN: Language Network; BGN: Basal Ganglia Network.

**Table S4**: Linear contents regression models examining relation of educational level and CSF biomarkers in aMCI and mild Alzheimer’s disease groups.

| **group** | **CR proxy** | **education** | | |
| --- | --- | --- | --- | --- |
|  | **CSF biomarker** |  |  |  |
|  |  | **β(SE)** | ***p*** |  |
| **aMCI** | t-tau | 0.170(0.009) | 0.371 |  |
|  | p-tau | 0.237(0.237) | 0.206 |  |
|  | Aβ_1-42_ | -0.2(6.509) | 0.409 |  |
| **Alzheimer’s** | t-tau | -0.131(0.017) | 0.668 |  |
|  | p-tau | -0.236(0.013) | 0.416 |  |
|  | Aβ_1-42_ | 2.372(62.617) | 0.410 |  |

Notes: aMCI: amnestic mild cognitively impaired subjects; CSF: cerebrospinal fluid; CR: cognitive reserve; β: linear regression coefficient; SE: standard error.

**REFERENCES**

Christensen, A.-L. (1975). Luria’s Neuropsychological Investigation, Manual and Test Material (4th ed ed.). Copenhagen: Munksgaard.

Christensen , P., & Guilford , J. (1959). *Manual for the Christensen Guilford Fluency Tests. (2nd ed.)*. Beverly Hills, California: Sheridan Supply.

Folstein, M. F., Folstein, S. E., & McHugh, P. R. (1975). "Mini-mental state". A practical method for grading the cognitive state of patients for the clinician. In *J Psychiatr Res* (Vol. 12, pp. 189-198). England.

Kaplan, E., Goodglass, H., & Weintraub, S. (1983). The Boston Naming Test. In L. Febiger (Ed.), (2nd edition ed.). Philadelphia.

Malloy-Diniz, L. F., Lasmar, V. A., Gazinelli Lde, S., Fuentes, D., & Salgado, J. V. (2007). The Rey Auditory-Verbal Learning Test: applicability for the Brazilian elderly. *Rev Bras Psiquiatr, 29*(4), 324-329.

Osterrieth, P. (1944). The test of copying a complex figure: A contribution to the study of perception and memory *Arch Psychol 30*, 206 –356.

Ratcliff, G. (1979). Spatial thought, mental rotation and the right cerebral hemisphere. *Neuropsychologia, 17*(1), 49-54.

Stroop, J. ( 1935). Studies of interference in serial verbal reactions. *Journal of Experimental Psychology., 18*, 643–662.

Sunderland, T., Hill, J. L., Mellow, A. M., Lawlor, B. A., Gundersheimer, J., Newhouse, P. A., et al. (1989). Clock drawing in Alzheimer's disease. A novel measure of dementia severity. *J Am Geriatr Soc, 37*(8), 725-729.
